# ApoB100-LDL Acts as a Metabolic Signal from Liver to Peripheral Fat Causing Inhibition of Lipolysis in Adipocytes

**DOI:** 10.1371/journal.pone.0003771

**Published:** 2008-11-20

**Authors:** Josefin Skogsberg, Andrea Dicker, Mikael Rydén, Gaby Åström, Roland Nilsson, Hasanuzzaman Bhuiyan, Sigurd Vitols, Aline Mairal, Dominique Langin, Peteris Alberts, Erik Walum, Jesper Tegnér, Anders Hamsten, Peter Arner, Johan Björkegren

**Affiliations:** 1 The Computational Medicine Group, Karolinska Institutet, Karolinska University Hospital, Solna, Stockholm, Sweden; 2 Department of Medicine, Karolinska Institutet, Karolinska University Hospital, Huddinge, Sweden; 3 Computational Biology Unit, Department of Physics, Linköping University, Linköping, Sweden; 4 Division of Clinical Pharmacology, Department of Medicine, Karolinska Institutet, Karolinska University Hospital, Solna, Stockholm, Sweden; 5 Inserm, U586, Obesity Research Unit, Toulouse, France; 6 Biovitrum AB, Stockholm, Sweden; 7 Atherosclerosis Research Unit, Department of Medicine, Karolinska Institutet, Karolinska University Hospital, Solna, Stockholm, Sweden; University of Parma, Italy

## Abstract

**Background:**

Free fatty acids released from adipose tissue affect the synthesis of apolipoprotein B-containing lipoproteins and glucose metabolism in the liver. Whether there also exists a reciprocal metabolic arm affecting energy metabolism in white adipose tissue is unknown.

**Methods and Findings:**

We investigated the effects of apoB-containing lipoproteins on catecholamine-induced lipolysis in adipocytes from subcutaneous fat cells of obese but otherwise healthy men, fat pads from mice with plasma lipoproteins containing high or intermediate levels of apoB100 or no apoB100, primary cultured adipocytes, and 3T3-L1 cells. In subcutaneous fat cells, the rate of lipolysis was inversely related to plasma apoB levels. In human primary adipocytes, LDL inhibited lipolysis in a concentration-dependent fashion. In contrast, VLDL had no effect. Lipolysis was increased in fat pads from mice lacking plasma apoB100, reduced in apoB100-only mice, and intermediate in wild-type mice. Mice lacking apoB100 also had higher oxygen consumption and lipid oxidation. In 3T3-L1 cells, apoB100-containing lipoproteins inhibited lipolysis in a dose-dependent fashion, but lipoproteins containing apoB48 had no effect. ApoB100-LDL mediated inhibition of lipolysis was abolished in fat pads of mice deficient in the LDL receptor (*Ldlr^−/−^Apob* ^100/100^).

**Conclusions:**

Our results show that the binding of apoB100-LDL to adipocytes via the LDL receptor inhibits intracellular noradrenaline-induced lipolysis in adipocytes. Thus, apoB100-LDL is a novel signaling molecule from the liver to peripheral fat deposits that may be an important link between atherogenic dyslipidemias and facets of the metabolic syndrome.

## Introduction

Lipoproteins containing apolipoprotein (apo) B are synthesized in the liver (apoB100-very low density lipoproteins (VLDL)) or the intestine (apoB48-chylomicrons). These particles are released into the circulation, where they undergo hydrolysis by lipoprotein lipase, generating free fatty acids that are taken up by peripheral organs such as the adipose tissue. The hydrolysis also generates atherogenic remnants such as, apoB100-low density lipoprotein (LDL), a major risk factor for atherosclerosis [1].

The intracellular lipolysis of adipocytes is believed to play a central role in regulating whole-body energy homeostasis [2], affecting both the release of free fatty acids to the circulation and the extent of peripheral fat deposits. These effects provide a metabolic link between peripheral fat deposits and the liver that influence the hepatic synthesis and secretion of lipoproteins [2]. However, the existence and possible nature of a reciprocal signal from the liver to the adipocytes have not been established.

The size and binding properties of apoB-containing lipoproteins imply that they may have physiological functions other than the passive transport of lipids to adipocytes. Indeed, individuals with conditions characterized by high levels of apoB-containing lipoproteins have reduced levels of catecholamine-induced lipolysis in their adipocytes [3], [4].

We hypothesized that apoB-containing lipoproteins affect adipocyte lipolysis, serving as a signal from the liver to peripheral fat deposits. To test this hypothesis, we investigated lipolysis in subcutaneous fat cells from obese but otherwise healthy men in relation to their levels of apoB-containing lipoproteins. We then examined noradrenaline-induced lipolysis in genetically modified mouse models with plasma containing high or intermediate levels or no apoB100 or apoB48, and human primary cultured adipocytes and 3T3—L1 cells incubated with different types and concentrations of human and mouse apoB-containing lipoproteins. Taken together, our findings show that apoB100-LDL act as a metabolic signal causing inhibition of lipolysis in adipocytes.

## Results

### Lipolysis in healthy volunteers and human primary adipocytes

**Figure 1:**
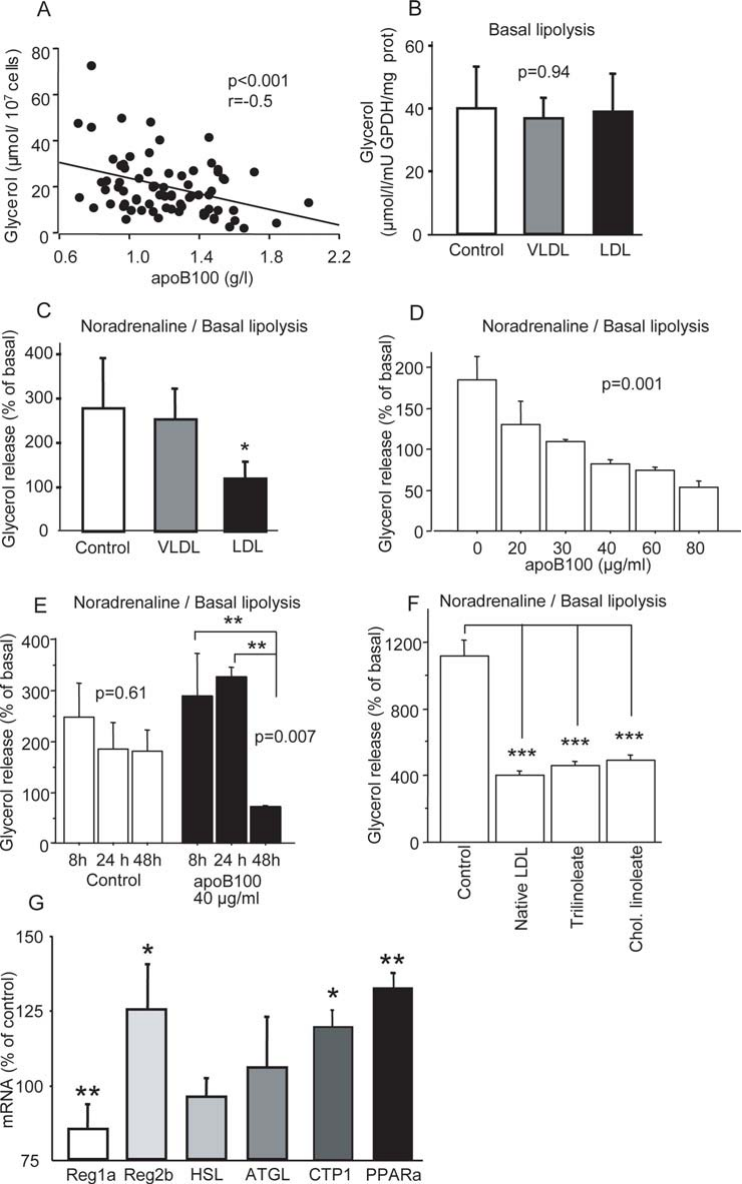
Effects of apoB100, VLDL, and LDL on lipolysis in human fat cells. (A) Linear regression plot of fat cell lipolysis (glycerol release) and plasma apoB100 levels in 48 overweight but healthy men. The linear regression r-value = −0.5, p<0.001. (B) Basal (n = 9; p = 0.94; DF = 2,24; F-value = 0.065) and (C) noradrenaline-stimulated (noradrenaline/basal) (n = 9; p<0.05; DF = 2,24; F-value = 3.6) lipolysis in human differentiated pre-adipocytes incubated with equal concentrations (40 µg apoB100/ml medium) of human VLDL or LDL or with vehicle alone for 48 hours. (D) Inhibition of lipolysis in human differentiated pre-adipocytes incubated without or with human LDL at five concentrations (20, 30, 40, 60, 80 µg apoB100/ml medium) for 48 hours (n = 3 for each concentration; p = 0.001; DF = 2,5,10; F-value = 10.4) and (E) with different incubation times (8, 24 and 48 hours) with 40 µg apoB100/ml medium (n = 5 for each time point; DF = 2,12) p = 0.61; F-value = 0.48 for vehicle and p = 0.007; F-value = 7.6 for apoB100 incubation. (F) Noradrenaline-stimulated (noradrenaline/basal) lipolysis in human differentiated pre-adipocytes incubated with vehicle alone, native LDL and two forms of reconstituted LDL (40 µg apoB100/ml medium) for 48 hours (n = 29–34 in each group). “Trilinoleate” indicates reconstituted LDL in which cholesterol esters had been replaced with Trilinoleate. “Chol. linoleate” indicates reconstituted LDL in which cholesterol esters had been replaced with cholesteryl linoleate. (G) mRNA expression (n = 4–8) in human differentiated pre-adipocytes incubated with human LDL (40 µg apoB100/ml medium) presented as % of control cells (not exposed to LDL). Error bars in (B,C,G) indicate SD and in (D,E,F) indicate SE. p-values for (B, C, D, E) were determined by ANOVA and for (F,G) un paired t-test. *p<0.05, **p<0.01, ***p<0.001 indicate Fisher's Post-hoc test.

In accordance with previous observations [3], [4], the rate of noradrenaline-induced lipolysis in subcutaneous fat cells from obese but otherwise healthy men correlated strongly and inversely with apoB plasma levels (r = −0.5, Fig. 1A) but was not related to apoA1 or body mass index (both r = 0.1; not shown), suggesting that apoB100-containing lipoproteins suppresses adipocyte lipolysis. The inverse correlation was also found to be independent of age and markers of the metabolic syndrome. This was investigated by multiple regression analysis using the following markers for metabolic syndrome and noradrenaline-induced lipolysis together as regressors versus apoB100 as a dependent factor, age; r = −0.27, p = 0.018; waist: r = −0.35, p = 0.005; fasting plasma insulin: r = −0.36, p = 0.002; fasting plasma HDL cholesterol: r = −0.36, p = 0.002; fasting plasma triglycerides: r = −0.32; p = 0.003; systolic blood pressure: r = −0.26, p = 0.023; diastolic blood pressure: r = −0.24, p = 0.032.

To determine the cause-effect relationship, we differentiated human preadipocytes into adipocytes and exposed them to equal numbers of human VLDL or LDL particles (i.e., equal concentrations of apoB100). Spontaneous (basal) lipolysis was unaffected (Fig. 1B); however, noradrenaline-induced lipolysis was markedly inhibited by LDL, but not by VLDL (Fig. 1C), in a concentration-dependent fashion (Fig. 1D) after incubation for 48 hours (Fig. 1E).

Reconstitution of the cholesterol esters content of the native LDL particles with triglycerides or cholesterol ester (experimental control) did not alter the inhibition of noradrenaline-induced lipolysis (Fig. 1F).

In our previous studies of lipolysis in hyper-apoB100 conditions, noradrenaline-induced lipolysis was attenuated at the level of the protein kinase A (PKA)–hormone sensitive lipase (HSL) complex [3], [4]. RT-PCR analyses of human adipocytes incubated with LDL revealed that the mRNA levels of the regulatory 1α subunit of the PKA were decreased (Reg1α; p<0.01), whereas that of the regulatory 2β subunit were increased (Reg 2β; p<0.05). LDL incubation did not influence the mRNA levels of HSL or adipose triglyceride lipase (ATGL) [5]. In the fatty acid oxidation pathway, the mRNA levels of carnitine palmitoyltransferase 1 (CPT1) and peroxisome proliferator activated receptor alpha (PPARα) were increased, p<0.05 and p<0.01, respectively (Fig. 1G).

### Lipolysis in Fat Pads in Relation to Plasma apoB in Mouse

**Figure 2:**
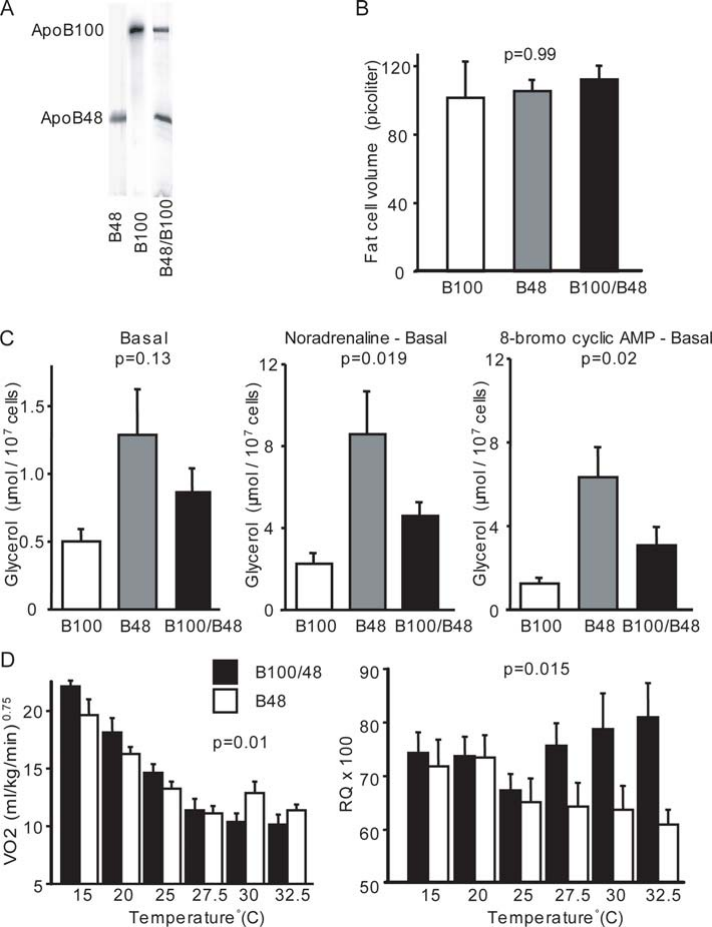
Effects of apoB100 in mice. Plasma and gonadal fat pads were isolated after an overnight fast from 10-week-old wild-type mice (B100/B48) and mice lacking apoB100 (B48) or expressing only apoB100 (B100). (A) Western blots of plasma performed with rabbit anti-mouse apoB antibodies. (B) Gonadal fat pad cell volume (n≥12 mice per group (B100/B48;B48; B100); p = 0.99; DF = 2,35; F-value = 0.015). (C) Basal (p = 0.13; DF = 2,39; F-value = 2.4), noradrenaline-stimulated (p = 0.019; DF = 2,36; F-value = 3.8), and 8-bromo cyclic AMP–stimulated lipolysis (p = 0.02; DF = 2,38; F-value = 3.8) (n≥12 mice per group). (D) Resting oxygen consumption (VO_2_) and respiratory quotient (RQ) in mice kept at different environmental temperatures (n = 7–8 mice mice per group; DF = 1,13) p = 0.01; F-value = 3.3 for VO_2_ and p = 0.015; F-value = 3.1 for RQ). Error bars in (B–D) indicate SD. p-values were determined by ANOVA.

To assess the role of different types of apoB-containing lipoproteins *in vivo*, we investigated fat pad lipolysis in mice that conditionally lack microsomal triglyceride transfer protein in the liver (*Mttp* ^Δ/Δ^) [6] (and therefore have no apoB100-containing lipoproteins in plasma), in wild-type mice with both apoB100 and apoB48, and in mice expressing only apoB100 [7] (Fig. 2A). These groups exhibited no differences in fat cell size (Fig. 2B), body weight (Table 1), or basal lipolysis (Fig. 2C). However, lipolysis induced by noradrenaline (at the maximal effective concentration) or 8-bromo cyclic AMP was increased in mice lacking apoB100, reduced in apoB100-only mice, and intermediate in wild-type mice (Fig. 2C). Dose-response experiments showed similar hormone sensitivities in all three groups (not shown).

**Table 1:** Basic Characteristics of the Study Mice.

	Genotype		
	ApoB100	ApoB100/48	ApoB48
	(*Apob^100/100^*)	(*wild-type*)	(*Mttp^Δ/Δ^*)
Characteristics	(*n* = 5)	(*n* = 5)	(*n* = 5)
Age (weeks)	10	10	10
Body weight (g)	27.1±0.57	29.4±2.6	29.7±2.0
Liver weight (g)	1.5±0.1^*^	1.3±0.05	1.5±0.09^†^
Plasma triglycerides (mg/dl)	33.1±10.6*	67.8±29.6	17.9±7.0^*^
Plasma cholesterol (mg/dl)	19.1±14.6^†^	99.1±16.5	38.5±13.7^†^
Plasma glucose (mg/dl)	361±42	373±20	360±31

Values are mean±SD. ^*^p<0.05, ^†^p<0.005 vs. apoB100/48 mice. Plasma were isolated after an overnight fast.

Of note, mice lacking apoB100 and apoB100-only mice had lower plasma triglyceride and cholesterol levels than wild-type mice (Table 1), implying that plasma concentrations of cholesterol and triglycerides *per se* did not affect lipolysis in adipocytes. The increased lipolysis in mice lacking apoB100 did not lead to a leaner phenotype or to smaller fat cells, suggesting adaptive or parallel changes in whole-body energy metabolism. Indeed, oxygen consumption was increased (VO_2_, Fig. 2D) in these mice—but only at high temperatures, when lipid oxidation was concomitantly increased, as evidenced by a lower respiratory quotient (RQ, Fig. 2D). However, food intake was almost identical in both groups (Fig. S1, Supplementary online material). These results suggest that mice lacking apoB100 decrease their circulating lipids rather than body fat as a result of the increase in catecholamine-induced lipolysis by enhancing energy expenditure and fat oxidation.

### Types of apoB and lipolysis in mouse adipocytes

**Figure 3:**
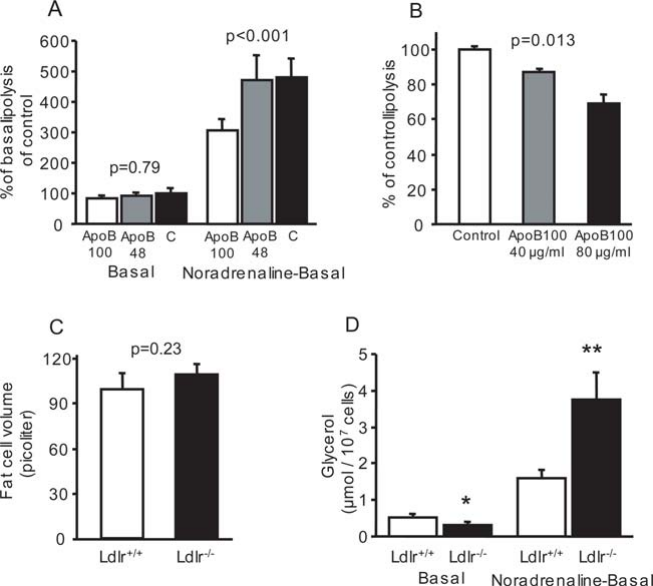
Effects of mouse apoB100 on 3T3-L1 cells and effects of Ldlr in the study mice. (A) Basal and noradrenaline-stimulated lipolysis in 3T3-L1 cells incubated with equal numbers of mouse lipoproteins containing only apoB100 (40 µg/ml medium, n = 10) or apoB48 (20 µg/ml medium, n = 4) or with vehicle alone (n = 7) for 48 hours; DF = 2,18 (p = 0.79; F-value = 0.24 for basal and p<0.001; F-value = 10.4 for noradrenaline-stimulated lipolysis). (B) Percent inhibition of lipolysis mediated by incubation of 3T3-L1 cells with mouse lipoproteins containing apoB100 at high (80 µg apoB/ml medium) and low (40 µg apoB/ml medium) concentrations or with vehicle alone (Control) (n = 9 for each concentration; p = 0.013; DF = 2,24; F-value = 5.6) for 48 hours. (C) Fat cell volume in gonadal fat pads isolated from *Ldlr* ^−/−^ *Apob* ^100/100^ mice (Ldlr^−/−^, n = 13) and *Ldlr* ^+/+^ *Apob* ^100/100^ mice (Ldlr^+/+^, n = 17) mice (p = 0.23). (D) Basal (p<0.05) and noradrenaline-stimulated (p<0.01) lipolysis in the mice described in (C). Error bars indicate SD. p-values were determined by ANOVA in (A–B) and unpaired t-test in (C–D), *p<0.05, **p<0.01 (*t*-test).

Our mouse *in vivo* studies implied that apoB100-containing but not apoB48-containing lipoproteins affect noradrenaline-induced lipolysis in adipocytes. To explore this possibility further, we exposed 3T3-L1 adipocytes to equal amounts of mouse lipoproteins containing either apoB100 or apoB48. ApoB100 lipoproteins inhibited noradrenaline-induced but not basal lipolysis (Fig. 3A) in dose-dependent fashion (Fig. 3B). ApoB48 lipoproteins did not affect lipolysis (Fig. 3A). Thus, lipoproteins containing apoB100, but not those containing apoB48, are responsible for the interaction with adipocytes, leading to reduced lipolysis within these cells.

### The role of the LDL receptor on adipocytes

Since the LDL receptor (LDLR) is expressed on adipocytes [8], we investigated its role in the interaction between apoB100 lipoproteins and adipocytes by analyzing apoB100-only mice that expressed the LDLR (*Ldlr* ^+/+^ *Apob* ^100/100^) or were LDLR-deficient (*Ldlr^−/−^Apob* ^100/100^) [9]. Apart from their plasma cholesterol levels, the two groups were phenotypically similar (values not shown). LDLR deficiency did not affect fat cell size (Fig. 3C) but decreased basal lipolysis slightly (Fig. 3D). Importantly, however, noradrenaline-induced glycerol release was almost threefold higher in *Ldlr^−/−^Apob* ^100/100^ mice (Fig. 3D), suggesting that binding of apoB100-LDL to the LDLR is necessary to mediate the inhibition of lipolysis in adipocytes.

## Discussion

ApoB100 is the main cholesterol transporter in the circulation and has a central role in atherogenesis. In this study, we show that plasma apoB100-containing lipoproteins interact directly with adipocytes, resulting in lower levels of catecholamine-induced lipolysis. VLDL and apoB48-containing lipoproteins had no effect on lipolysis, indicating that the interaction with adipocytes requires the LDLR. We also provide evidence that apoB100-LDL modulates lipid mobilization and energy expenditure. These findings reveal a novel role for apoB100-LDL as a signaling molecule between the liver and adipose tissues that may provide an important link between atherogenic dyslipidemias and the metabolic syndrome.

**Figure 4:**
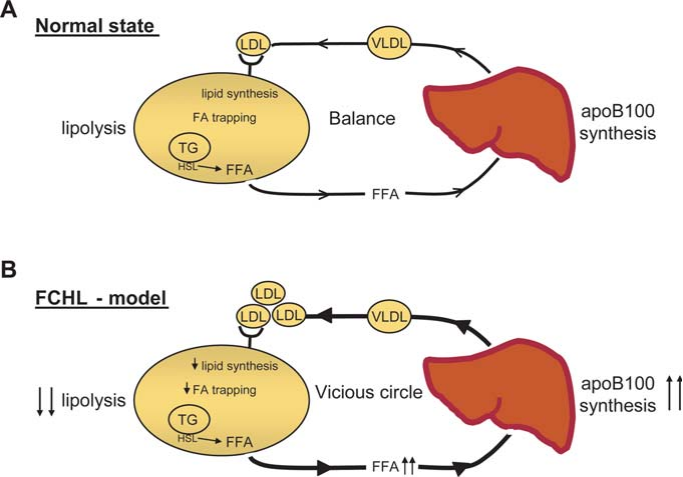
Model for cross-talk between the liver and adipose-tissue. In the normal state there is a balance between production of hepatic apoB100 containing lipoproteins and release of free fatty acid (FFA) from the adipose tissue. In FCHL patients, who have lower noradrenaline-induced lipolysis and increased levels of plasma lipoproteins containing aoB100, lowered lipid synthesis and decreased adipocyte fatty acid trapping lead to elevated levels of FFA in the circulation. Increased levels of circulating FFA activate apoB100 synthesis leading to elevated plasma apoB100 containing lipoprotein levels. In this fashion an imbalance between liver-adipose tissue is created and a “vicious circle” evolves. HSL indicates hormone sensitive lipase.

A role of apoB100-LDL as a liver–adipose signal is consistent with the notion that the liver is a major regulator of whole-body energy homeostasis and that white adipose tissue is an important energy reserve [10]. Indeed, fatty acids function as signaling molecules in the reverse direction, from the adipose tissue to the liver. During sympathetic activation, white adipose tissue releases fatty acids into the circulation at higher rates, at least in part through catecholamine-mediated activation of protein kinase A. This stimulates lipid oxidation and energy expenditure. The liver responds by increasing VLDL synthesis (Fig. 4A). The physiological significance of the observed correlation between plasma apoB100 levels and fat cell lipolysis (Fig. 1A) needs, however, to be interpreted with caution. For instance, this correlation could also be a consequence of one or several differential expressed genes with independent effects on both apoB100 plasma levels (either by affecting synthesis in the liver or clearance in the periphery) and lipolysis in fat cells.

Our findings suggest that decreased LDL concentrations in the microcirculation of adipose tissue cause a reciprocal activation of lipolysis in white adipose tissue when apoB100-LDL levels are decreased. In FCHL and metabolic syndrome, which are characterized by very high levels of apoB100 [11], a liver–adipose tissue route could potentially be involved. We speculate that, when catecholamine-induced adipocyte lipolysis decreases [3], [4], adipocyte lipid synthesis is lowered to compensate for the reduced lipolysis. In support of this notion, lipid synthesis stimulation by acylation-stimulating protein is decreased in FCHL [12], [13]. Patients with FCHL have increased apoB100 levels and decreasing catecholamine-induced lipolysis but the fat cell size remains normal, suggesting a link between low lipolysis and low lipid synthesis in fat cells. Reduced turnover of lipids in fat cells [3] may lead to decreased adipocyte fatty acid trapping and elevated levels of circulating fatty acids, which in turn exacerbate the dyslipidemia [14], [15] (Fig. 4B).

There are numerous possibilities for how the interaction between apoB100-LDL and adipocyte LDLR may influence catecholamine signaling to the adipose tissue. The LDLR itself may mediate second signaling by mediating the uptake and degradation of LDL particles, which in turn may release molecules with signaling properties [16], [17]. We tested whether the cholesterol ester content of the apoB100-LDL particles may alter their inhibitory effects on catecholamine-induced lipolysis. However, reconstituting cholesterol esters of the apoB100-LDL particles with triglycerides had no effect (Fig. 1F). Despite this, we found that apoB100-LDL signaling through the LDLR altered gene expression of several genes whereof two in the PKA complex (Fig. 1G). The gene expression observations are consistent with the results of previous studies: first, the documented changes in Reg1α and Reg2β in Reg1α-null mutant mice and the findings of lipolysis studies with selective PKA blockers in mouse fat cells [18]–[20] are in agreement with a decrease in catecholamine-induced lipolysis. Furthermore, knockout of Reg2β results in a compensatory increase in Reg1α [18], [19]. The induction of apoB100-LDL of genes regulating fatty acid oxidation (CPT1, PPARα) are, in our view, in agreement with the measurements of metabolic rates (VO_2_ and RQ, Fig. 2D). A decrease in catecholamine-induced lipolysis seems to be coupled with an increase in fatty acid oxidation, thus the two phenomena appear to compensate each other which also is consistent with the absence of changes in fat cell volumes (Fig. 2B). However, the exact mechanism for how the LDLR/apoB100-LDL complex mediates changes to gene expression and inhibitory effect on lipolysis remains a topic for future studies.

In conclusion, we have shown that apoB100-LDL mediates the inhibition of catecholamine-induced lipolysis in adipocytes through the LDLR. Our findings suggest a novel link between dyslipidemias and metabolic disorders such as FCHL and the metabolic syndrome. They also suggest that the beneficial actions of cholesterol-lowering agents such as statins may involve effects on the rate of lipolysis in adipocytes.

## Materials and Methods

### Subjects

The study subjects were 48 overweight but otherwise healthy Scandinavian men (body mass index, 25–53 kg/m^2^), aged 23–72 years, who were free from regular medication. In the morning after an overnight fast, a venous blood sample was obtained for analysis of serum apoB100 and apoA1 concentrations, and a subcutaneous fat biopsy (1–3 g) was obtained from the abdominal region by needle aspiration under local anesthesia as described [21]. For experiments in primary cultures of human pre-adipocytes, subcutaneous adipose tissue was obtained during cosmetic liposuction under general anesthesia from healthy subjects who were not selected on the basis of age, sex, or degree of obesity. The study was approved by the hospital's ethics committee. The study was explained in detail to each participant and written informed consent was obtained.

### Mouse models

In the *Mttp* ^flox/flox^Mx1-*Cre^+/−^* mice used for the study, the gene for microsomal triglyceride transfer protein is floxed (*Mttp* ^flox/flox^) and can be recombined in the liver upon induction of Cre-recombinase (Mx1-*Cre^+/−^*) with polyinosinic-polycytidylic ribonucleic acid (1 µg/µl; Sigma). This recombination results in the termination of hepatic VLDL synthesis and depletion of plasma apoB100, as described [6]. Two weeks before sacrifice, *Mttp* was recombined (*Mttp* ^Δ/Δ^), which interrupts hepatic synthesis of apoB100-containing lipoproteins, leaving only apoB48-containing lipoproteins in plasma (Fig. 2A). Littermate wild-type mice (*Mttp* ^flox/flox^), injected with phosphate-buffered saline, had approximately equal concentrations of plasma apoB100 and apoB48, as shown by western blot analysis, while *Apob* ^100/100^ mice [7] only had apoB100 in plasma (Fig. 2A). *Ldlr* ^−/−^ *Apob* ^100/100^ and *Ldlr* ^+/+^ *Apob* ^100/100^ mice were generated by crossing *Ldlr* ^−/−^ mice [9] with *Apob* ^100/100^ mice. The study mice had been backcrossed at least six times (>95% C57BL/6, <5% 129/SvJae), were housed in a pathogen-free barrier facility (12 h light/12 h dark cycle), and were fed rodent chow containing 4% fat. Genotypes were determined by polymerase chain reaction using genomic DNA from tail biopsies. Mice were sacrificed at 10 weeks of age. Gonadal and renal fat pads were immediately removed and put into tubes containing 37°C phosphate-buffered saline for following analyses.

### Mouse characteristics and metabolic rate

Mouse plasma samples (2 µl) were fractionated by SDS-PAGE using 4% gels. The apoB proteins were detected with rabbit antiserum against mouse apoB (Biosite, Taby, Sweden). The binding of primary antibodies was assessed with horseradish peroxidase–labeled donkey anti-rabbit antibodies and ECL western blotting reagents (Amersham Biosciences, GE Healthcare Biosciences, Little Chalfont, Buckinghamshire, England). Plasma cholesterol and triglyceride concentrations were determined with colorimetric assays (Infinity cholesterol/triglyceride kits; Thermo Electron, Melbourne, Australia). Plasma glucose levels were measured with Precision Xtra (MediScience, Cherry Hill, NJ).

Eight mice lacking apoB100 (*Mttp^Δ/Δ^*) and seven wild-type (*Mttp* ^flox/flox^) mice were investigated in metabolic cages, and food intake was recorded. Energy expenditure and respiratory quotient were determined at week 10 as described [22]. In brief, after an acclimatization period, oxygen consumption and carbon dioxide production were measured at 15–32.5°C. Experiments conducted at temperatures of 15–27.5°C lasted for up to 5 hours; those conducted at temperatures above 27.5°C lasted for 2.5 hours (to avoid stress caused by the heat). Oxygen consumption (ml/min/kg^0.75^) and the respiratory quotient were calculated with a calorimetry calculation program (written in DasyLab; Somedic, Horby, Sweden).

### RNA Isolation and Real-Time PCR

Human subcutaneous fat that had been stored in Trizol reagent at −80°C were homogenized (FastPrep, Qbiogene; Irvine, CA) in fresh Trizol. Total RNA was prepared with RNeasy mini kits (Qiagen, Valencia, CA) using a DNAse I treatment step according to the manufacturer's protocol. RNA quality was assessed with a Bioanalyzer 2100 system (Agilent Technologies, Santa Clara, CA). Total RNA (0.3–0.5 µg) was reverse transcribed with Superscript II as recommended by the manufacturer (Invitrogen). After dilution to 65 µl, cDNA (3 µl) was amplified by real-time PCR with 1× TaqMan universal PCR master mix (Applied Biosystems, Foster City, CA). Assay-on-Demand kits containing corresponding primers and probes were from Applied Biosystems. The mRNA levels were normalized to acidic ribosomal phosphoprotein P0 (RPLP0).

### Lipoprotein isolation

Human VLDL and LDL in 60 ml of blood from a normolipidemic donor were isolated by density-gradient ultracentrifugation, as described [23]. In brief, after a 16-hour spin in a Beckman SW40 rotor at 40,000 rpm at 15°C, the top 0.5 ml layer was aspirated (VLDL). The tubes were then sliced 42 mm from the top to harvest the LDL fraction. The LDL and VLDL fractions were each passed through a PD-10 desalting column (Amersham Bioscience). Mouse lipoproteins containing apoB48 or apoB100 were isolated from plasma pooled from 8 *Mttp^Δ/Δ^* mice and 8 *Apob* ^100/100^ mice, respectively. The pooled plasma was mixed with 1.42 g/ml NaBr to a final density of 1.10 g/ml and ultracentrifuged at 40,000 rpm in a Beckman SW40 rotor for >80 hours at 10°C. The top 0.5 ml of the tube containing LDL and less dense lipoproteins was collected. ApoB concentrations in the lipoprotein fractions were assessed from total protein concentrations. The LDL fractions used in the current experiments contained 5.4±1.9 mmol/L cholesterol, 0.58±0.12 mmol/l triglycerides and 483±62 mg/l apoB100. We have run aliquots of these fractions on SDS-acrylamide gels showing a robust apoB100 band and no other degradation products or other proteins (data not shown).

### Preparation of reconstituted low density lipoprotein

Reconstituted LDL was prepared as described by [24]. In brief, LDL was dialyzed at 4°C against 0.3 mM EDTA, pH 7.0. Aliquots of 1.9 mg LDL were transferred to glass tubes containing 25 mg of potato starch and frozen in liquid nitrogen. The samples were lyophilized and the endogenous lipids were extracted from the lyophilized LDL with heptane. After removing the last heptane supernatant, 200 µl heptane containing 6 mg of glyceryl trilinoleate or cholesteryl linoleate (Sigma-Aldrich) were added. The mixture was incubated for 1 hour at 4°C followed by evaporation of the heptane under a gentle stream of nitrogen until the samples were completely dry. One ml of PBS, pH 7.4, was then added to dissolve the reconstituted LDL and left overnight at 4°C. The solubilized reconstituted LDL was separated from the bulk of the starch and from excess lipids by centrifugation at 2000 rpm for 10 min, 4°C. The supernatant was further centrifuged at 9100 g for 10 min, 4°C and finally filtered through a 0.22 µm Millipore filter.

### Adipose tissue and cell cultures

Human subcutaneous adipose tissue and mouse fat pads were cut into fragments, incubated with collagenase (0.5 g/ml; Sigma, St. Louis, MO) in Krebs Ringer phosphate buffer (pH 7.4) supplemented with 40 g/l dialyzed bovine serum albumin (fraction V; Sigma) for 1 hour at 37°C, and filtered through a nylon mesh. The supernatant, containing mature adipocytes, was used as described below.

Pre-adipocytes were isolated and differentiated as described [25]. The cell pellet obtained from human subcutaneous adipose tissue was resuspended in 10 ml of erythrocyte lysis buffer (0.154 M NH_4_Cl, 5.7 mM K_2_HPO_4_, and 0.1 mM EDTA, pH 7.3) and centrifuged. The pellet was resuspended in 10 ml of DMEM/F12 medium (Invitrogen) and filtered through a nylon filter. After an additional centrifugation, the cells were resuspended in DMEM/F12 supplemented with 10% fetal calf serum and penicillin/streptomycin (100 mg/l), seeded into 12-well plates (50,000 cells/cm^2^), and kept at 37°C in 5.3 kPa CO_2_ for 18–20 hours. Cells were then washed with DMEM/F12 and cultured in chemically defined serum-free medium. Rosiglitazone (kindly provided by SmithKline Beecham Pharmaceuticals) was added to a final concentration of 10 mmol/l (days 1–6) to induce differentiation. Cells were maintained in the medium at 37°C and 5.3 kPa CO_2_ for a total of 10 days and incubated with fresh lipoproteins for 8, 24, or 48 hours. Only cultures with a differentiation density ≥70% and <5% contaminating endothelial cells were used. Differentiation was determined by quantifying glycerol-3-phosphate dehydrogenase activity as described [25].

Standard procedures were used to grow and differentiate 3T3-L1 cells. Lipolysis experiments with differentiated 3T3-L1 cells or human pre-adipocytes were conducted as described [26]. In brief, pre-adipocytes were incubated in medium with or without fresh mouse or human lipoproteins for 48 hours. The medium was removed, and the cells were washed and incubated for 3 hours at 37°C in DMEM/F12 medium supplemented with bovine serum albumin (20 g/l) with and without noradrenaline (10^−4^ mol/l). In methodological experiments, 10^−4^ mol/l of noradrenaline always produced the maximal lipolytic effect. Aliquots were collected and kept at −20°C for measurement of glycerol concentration (an index of lipolysis). To control for interexperimental differences in adipocyte differentiation, glycerol release was normalized to the glycerol-3-phosphate dehydrogenase activity and protein content of each sample.

Mature collagenase-isolated fat cells were used for lipolysis experiments as described [3], [4]. The same lipolysis protocol was used for human and mouse adipocyte preparations. Lipolysis was measured in one of the two gonadal fat pads from each mouse. In preliminary experiments, similar results were obtained with gonadal fat pads and equal amounts of renal adipose tissue. Fat-cell size and number were measured (see below) [27]. In lipolysis experiments, diluted cell suspensions (2%, vol/vol) were incubated in albumin-containing buffer, pH 7.4, containing glucose (1 g/l) and ascorbic acid (0.1 g/l) for 2 hours at 37°C with air as the gas phase. Glycerol release into the medium was determined by bioluminescence as described [28] and expressed as mol ⋅ 10^−6^ ⋅ 2 h^−1^ ⋅ cells ⋅ 10^−7^. Adipocytes were incubated in the absence or presence of 10^−6^ mol/l noradrenaline and 10^−3^ mol/l 8-bromo cyclic AMP (a phosphodiesterase-insensitive cyclic AMP analogue). In the clinical experiments isolated human fat cells were also incubated with isoprenaline 10^−6^ mol/l (nonselective beta adrenergic agonist) and forskolin 10^−4^ mol/l (activator of adenylyl cyclase). These concentrations of lipolytic agents always yielded maximal stimulation in mature adipocytes in control experiments. When several hormone concentrations were used, the concentration-response curves were analyzed for half-maximum effective concentration.

### Measurement of fat cell size and volume

Mean fat cell size and volume were determined on isolated fat cells as follows: Fat cell diameter was measured in direct microscopy and averaging the diameter of 100 cells in each individual or mouse. The mean fat cell volume and size were calculated by formulas developed by Hirsch and Gallian [29]. Coefficient of variation for the method is 2–3%. The mean values are essentially the same as those determined from fat cell sizing of intact pieces of human adipose tissue [30]. Total number of fat cells in the body was calculated as the amount of body fat divided by the mean fat cell weight. It is well known that mean fat cell volume and weight differs between various adipose regions in man. However, the differences are small and only introduce a marginal error when just one depot is used for the calculation of total fat cell number as discussed [31].

### Statistical analyses

Values were compared with unpaired *t* tests, simple and multiple regression analyses, or analysis of variance (ANOVA) followed by Fisher's post-hoc test. Data with a skewed distribution were log normalized before statistical analyses.

## Supporting Information

Figure S1 Food intake in B100/48 (n = 7) and B48 (n = 8) mice. Error bars indicate SD.
(18.20 MB TIF)
