# Supplementary figures and images for: ApoB100-LDL Acts as a Metabolic Signal from Liver to Peripheral Fat Causing Inhibition of Lipolysis in Adipocytes
Source: PLoS One. 2008 Nov 20;3(11):e3771. doi: 10.1371/journal.pone.0003771 (PMC2582480; doi:10.1371/journal.pone.0003771)

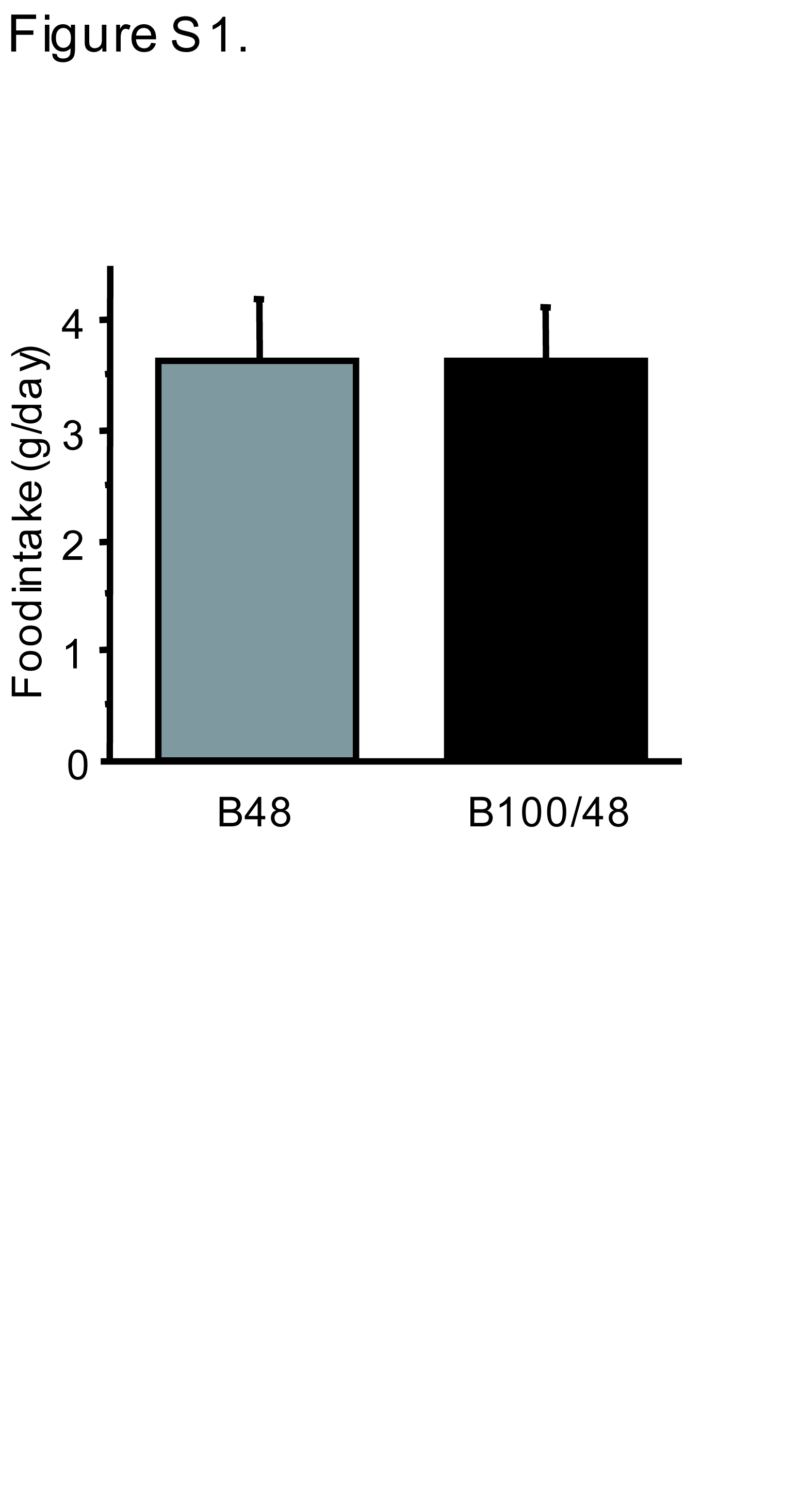

Supplement: Figure S1 — Food intake in B100/48 (n = 7) and B48 (n = 8) mice. Error bars indicate SD. (18.20 MB TIF) [file pone.0003771.s001.tif]
